# 814. Successful Treatment of *Cutibacterium* acnes (CA) Prosthetic Device Infection (PDI) with Oral Linezolid and Rifampin (LR)

**DOI:** 10.1093/ofid/ofab466.1010

**Published:** 2021-12-04

**Authors:** Ronald G Nahass, Maalikat Esquivel, Krystle Smith, Danielle Heinemann, Kathleen H Seneca

**Affiliations:** 1 ID Care, Hillsborough, New Jersey; 2 ID CARE, Hillsborough, New Jersey; 3 The College of New Jersey, Flemington, New Jersey

## Abstract

**Background:**

CA PDI is increasingly recognized. CA is felt to create a slime layer that makes infection more likely and treatment more difficult in this setting. Traditional management has included prosthetic device explantation (PDE), prolonged antibiotic treatment, and delayed reimplantation. Recent interest in the use of oral treatment regimens and single stage procedures with long duration antibiotic therapy led us to treat a series of patients with oral treatment and retained prosthesis after debridement. We report those results.

**Methods:**

Sequential patients with CA PDI treated with oral therapy were identified. All patients underwent debridement of the tissue, exchange of components and/or reimplantation of the prosthetic device. Only patients with exchanges were included. PDE was excluded. MIC testing for CA isolates was obtained when possible. Initial treatment was recorded at time of surgery. LR was the treatment of choice unless toxicity developed. A minimum of a 3-month follow-up post treatment was required to be included. 6 and 12 month follow up were obtained for all patients but 1 at this time.

**Results:**

10 patients were treated (Table 1). Shoulder joint infections were most common. All patients were treated with LR. All completed a minimum of 42 days of treatment (Table 2). The medication was well tolerated. The most common adverse events were nausea. 9/10 patients with 12 month follow up had no evidence of relapse. 1/10 had no relapse at 3 months. Typical for CA infection laboratory markers for infection were not markedly elevated. Notably thrombocytopenia did not occur (Table 3).

Table 1. Distribution of Prosthetic Device Infections

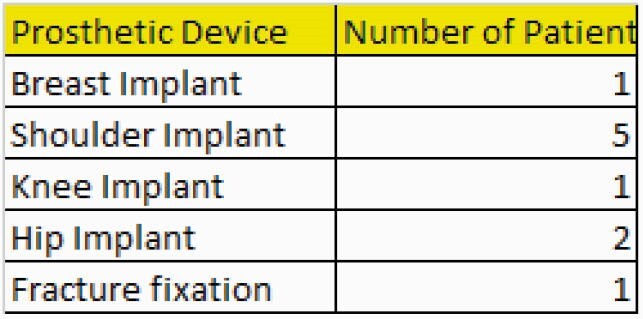

Table 2. Duration of Treatment

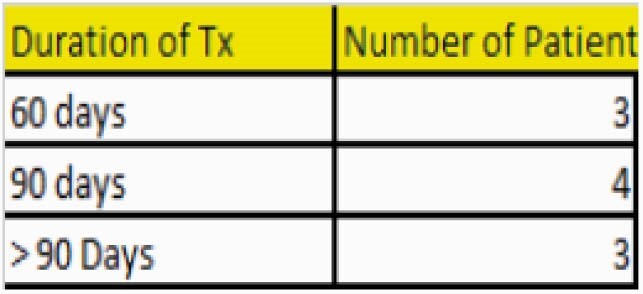

Table 3. Selected Laboratory Results

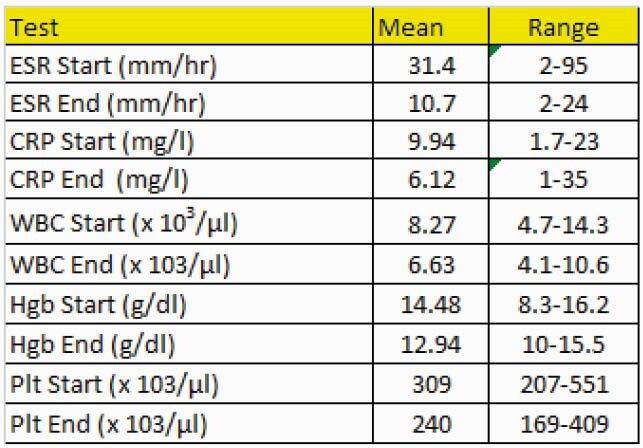

**Conclusion:**

We demonstrated the ability to successfully treat 10/10 patients with CA PDI without explantation using prolonged oral treatment with LR after debridement. This combination should be considered a treatment option and explored further as a low cost, well tolerated, high value treatment approach to this difficult infection.

**Disclosures:**

**Ronald G. Nahass, MD**, **Abbvie** (Grant/Research Support, Speaker’s Bureau)**Alkermes** (Grant/Research Support)**Gilead** (Grant/Research Support, Speaker’s Bureau)**Merck** (Grant/Research Support, Speaker’s Bureau) **Kathleen H. Seneca, MSN**, **Abbvie** (Research Grant or Support)**Alkermes** (Research Grant or Support)**Gilead** (Speaker’s Bureau)

